# High-grade pleomorphic sarcoma associated with an orthopedic implant: a rare case report

**DOI:** 10.1093/omcr/omad061

**Published:** 2023-06-26

**Authors:** Abdellatif Benabbouha, Youssef Benyass, Hicham Sallahi, Omar Margad

**Affiliations:** Department of Orthopedic Surgery and Traumatology, Military Training Hospital Avicenne, Faculty of Medicine and Pharmacy, University Cadi Ayyad, BP 40150 Marrakech, Morocco; Department of Orthopedic Surgery and Traumatology, Military Training Hospital Avicenne, Faculty of Medicine and Pharmacy, University Cadi Ayyad, BP 40150 Marrakech, Morocco; Department of Orthopedic Surgery and Traumatology, Military Training Hospital Avicenne, Faculty of Medicine and Pharmacy, University Cadi Ayyad, BP 40150 Marrakech, Morocco; Department of Orthopedic Surgery and Traumatology, Military Training Hospital Avicenne, Faculty of Medicine and Pharmacy, University Cadi Ayyad, BP 40150 Marrakech, Morocco

## Abstract

The use of prosthetic implants and metallic materials is widespread in modern orthopedic surgery. Generally, these materials are non-toxic and inert. Nevertheless, a few cases of malignancy associated with certain implants have been documented in the literature. It has been reported that some components of these implants have carcinogenic properties. In most cases, these tumors are high-grade sarcomas that occur in the bone or soft tissue adjacent to the implant site. Here we present the case of a 53-year-old patient who underwent intramedullary nailing of the tibia and developed a pleomorphic sarcoma at the implant site 18 years later.

## INTRODUCTION

Prosthetic implants and metallic hardware are widely used in modern orthopedic surgery. They have significantly improved patient outcomes. These materials are relatively inert and non-toxic. Nevertheless, some of their constituents may have a neoplastic potential [1]. Furthermore, the development of malignant tumors associated with certain implants is extremely rare. In the orthopedic literature, only a few cases have been documented [1–5]. We hereby report the case of a 53-year-old patient who underwent intramedullary nailing of the tibia and developed pleomorphic sarcoma at the site of implant 18 years later.

## CASE REPORT

A 53-year-old man presented to our orthopedic department with two rapidly growing, ulcerated masses on the left leg. The patient reported that these masses had been developing for 2 months and were painless. He also reported a progressive weight loss of 5 kg and intermittent fever.

**Figure 1:**
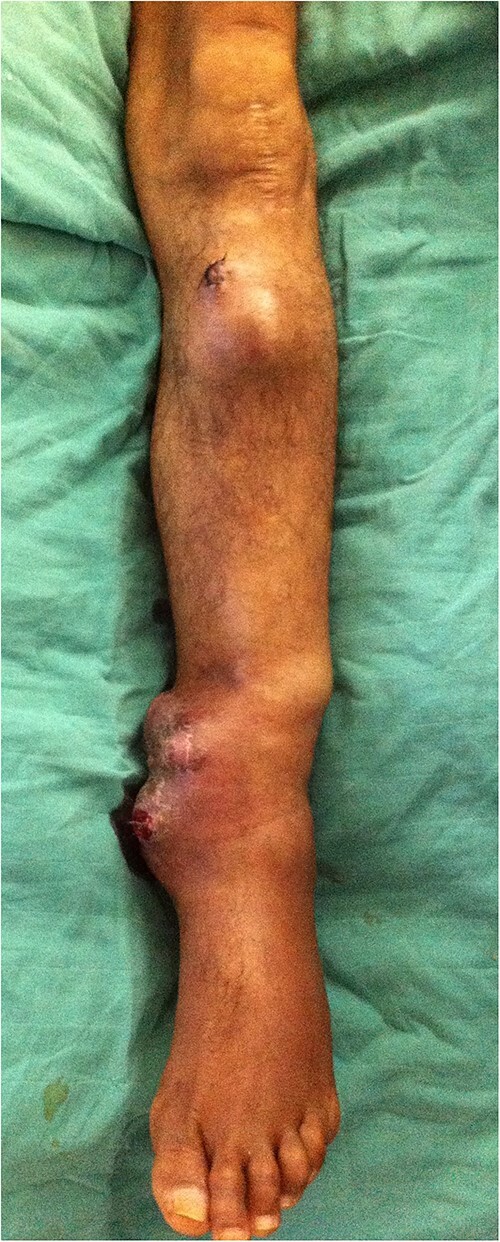
Two ulcerated masses on the left leg.

The patient’s medical history included a left tibial fracture surgically treated with intramedullary nailing 18 years ago. Upon physical examination, the first mass measured 10 cm in the anterior aspect of the upper leg, with no inflammatory signs opposite. However, the second mass measured 15 cm and was ulcerated with necrosis on the inferomedial aspect of the leg (Fig. 1). The lymph node examination was uneventful.

**Figure 2:**
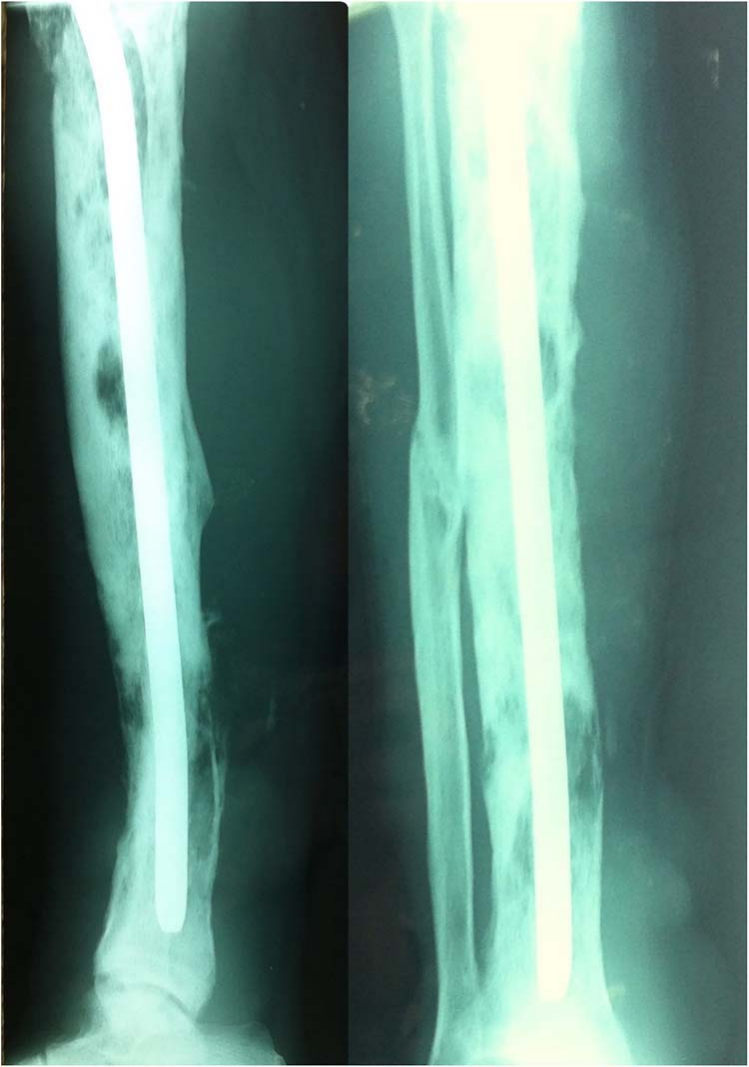
The X-ray showed a nail intramedullary in the left tibia, and multiple osteolytic lesions, heterogeneous, poorly limited with permeative destruction of the cortical bone.

**Figure 3:**
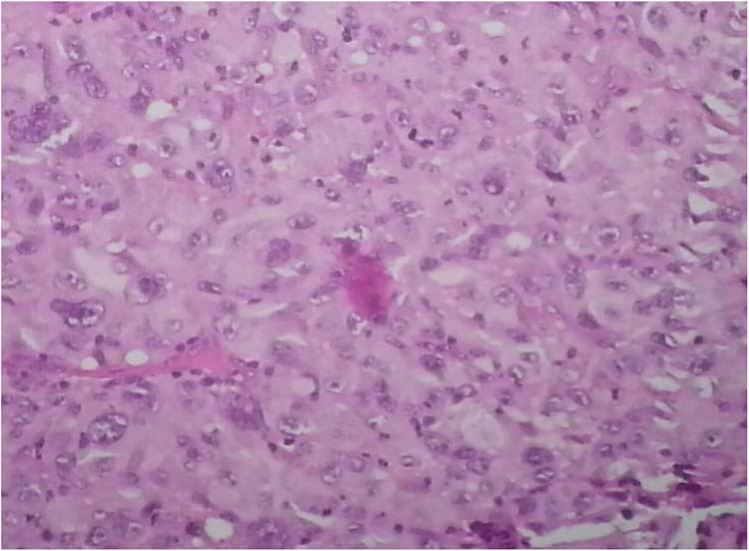
Histological examination revealed an undifferentiated high-grade pleomorphic sarcoma.

**Figure 4:**
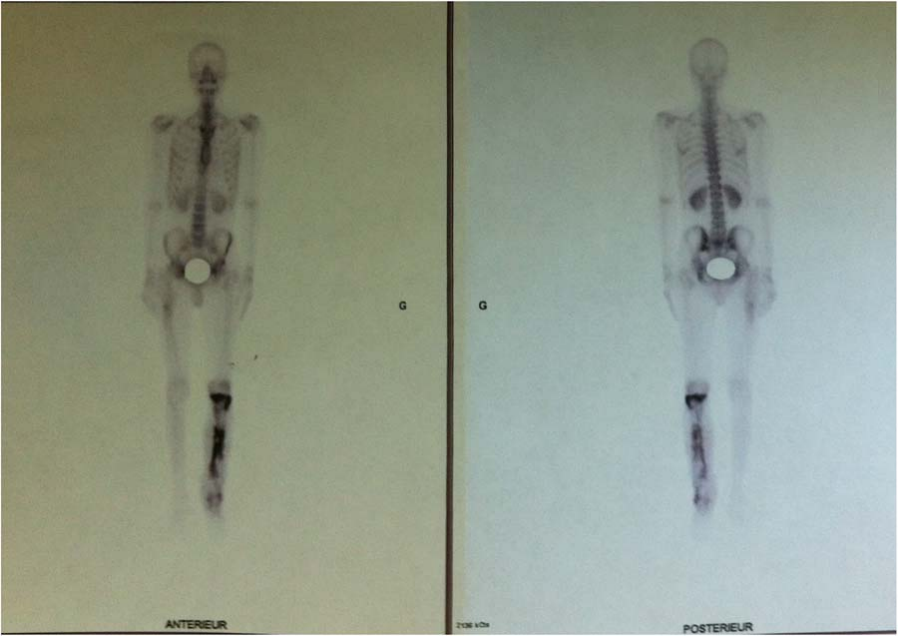
Bone scan showed increased tracer uptake only in the left tibia.

Anteroposterior and lateral radiographs of the left leg showed a nail intramedullary in the left tibia, and multiple osteolytic lesions, heterogeneous, poorly limited with permeative destruction of the cortical bone (Fig. 2). The metallic implant is incompatible with magnetic resonance imaging. Therefore, a biopsy of the first mass was taken, which revealed an undifferentiated high-grade pleomorphic sarcoma (Fig. 3). A bone scan was performed and showed increased tracer uptake only in the left tibia (Fig. 4). A whole-body computed tomography scan revealed metastases in the lungs.

Based on the past history, clinical, imaging and histologic findings, the diagnosis of high-grade undifferentiated pleomorphic sarcoma with bone involvement in association with an implanted orthopedic hardware was retained. Consequently, our multidisciplinary team of orthopedists, oncologists and pathologists discussed the different treatment options and, after obtaining the patient’s consent, we opted for radical treatment (transfemoral amputation) and adjuvant chemotherapy for metastasis. After receiving the first dose of chemotherapy, the patient was discharged from the hospital. The follow-up was initially scheduled every 3 months for 1 year, then every year after that. At 6 months follow-up, the patient tolerated the prosthesis well and responded favorably to chemotherapy. Three years later, he is alive and there are no distant metastases and no local recurrences.

## DISCUSSION

The occurrence of malignancy associated with implanted orthopedic prosthesis or hardware is extremely rare. Until now, approximately 50 cases of sarcoma associated with orthopedic implants have been reported in the literature [4]. As compared to the multitude of orthopedic procedures performed each year, this accounts a very small percentage of cases. Although the pathogenesis of this phenomenon is not yet clear, it has been reported in animal and human studies that some components of these implants, such as nickel, stainless steel, chromium, cobalt, iron, manganese, selenium, zinc and silicon have carcinogenic properties [1, 6]. In addition, other authors assume that implant-induced osteonecrosis contributes to malignancy development [7].

Implant-associated sarcomas were seen in patients of widely varying ages 11–87 years, with a mean age of 50 years [1]. There was no gender dominance. Usually, sarcomas develop between 6 months and 30 years following implant implantation, with a mean interval of 9 years [1]. In our case, the interval was 18 years. These sarcomas generally occur around the implant site in the bone or soft tissue adjacent. In terms of location, the femur is the most commonly affected bone, followed by the tibia, pelvis and humerus. In these locations, implanted material is quite frequent, which can explain this distribution [5]. Histopathology, these tumors are high-grade sarcomas including pleomorphic sarcoma, osteosarcoma, angiosarcoma, Ewing’s sarcoma, fibrosarcoma, epithelioid sarcoma, chondrosarcoma and synovial sarcoma. It seems that the type of sarcoma and the implanted material do not correlate [1].

High-grade undifferentiated pleomorphic sarcoma, formerly known as malignant fibrous histiocytoma, is a rare malignancy. It is usually seen in the 6th and 7th decades. About 75% of these tumors occur in the soft tissues of the extremities mainly in the lower limbs [8]. Typically, patients present with an enlarged, painless mass. In 5% of cases, metastases are found at initial presentation as seen in our patient. The primary treatment for these sarcomas is surgical resection en bloc with a margin of normal tissue of 1–2 cm around the tumor [9]. However, in some cases, particularly lower extremity sarcomas, amputation is preferred over difficult reconstruction. Moreover, prosthetic techniques have evolved significantly in recent years, resulting in improved tolerance and excellent performance [10]. Radiotherapy can reduce the risk of local recurrences whereas chemotherapy is usually reserved for metastatic disease [9].

Lastly, it is extremely rare for a malignant tumor to develop in association with an orthopedic implant. Nevertheless, physicians should be aware of this possibility, especially if new symptoms occur related to orthopedic implants.
